# Prevention of spina bifida: folic acid intake during pregnancy in Gulu district, northern Uganda

**DOI:** 10.11604/pamj.2015.20.90.5338

**Published:** 2015-01-30

**Authors:** Femke Bannink, Rita Larok, Peter Kirabira, Lieven Bauwens, Geert van Hove

**Affiliations:** 1 Ghent University, Faculty of Psychology and Educational Sciences, Ghent University Henry Dunantlaan 2, B 9000 Ghent, Belgium; 2 International Health Sciences University, Kampala, Uganda; 3 International Federation for Spina Bifida and Hydrocephalus, Cellebroersstraat 16 - 1000 Brussels, Belgium

**Keywords:** Folic acid, spina bifida, pregnancy, antenatal care, Uganda

## Abstract

**Introduction:**

The intake of folic acid before conception and during the first trimester of pregnancy can prevent spina bifida. This paper describes folic acid intake in women in Gulu district in northern Uganda.

**Methods:**

Structured interviews were held with 394 women attending antenatal care (ANC), 15 mothers of children with spina bifida, and 35 health workers in 2012 and 2013. SPSS16 was used for data analysis.

**Results:**

1/4 mothers of children with spina bifida took folic acid during late pregnancy, none preconception. None had knowledge about folic acid and spina bifida prevention. 33.5% of women attending ANC had ever heard about spina bifida, 1% knew folic acid intake can prevent spina bifida. 42.4% took folic acid supplements in late pregnancy, 8.1% during the first trimester, none preconception. All women said to have eaten food rich in folic acid. None were aware about fortified foods. 7% of health workers understood the importance of early folic acid intake. All health workers recommended folic acid intake to women attending ANC. 20% of the health workers and 25% of the women said folic acid supplements are not always available.

**Conclusion:**

Folic acid intake is limited in northern Uganda. This is attributed to limited education and understanding of women and health workers about the importance of early folic acid intake, late presentation of women at ANC, poor supply chain and dilapidated health services caused by war and poverty. A combination of food fortification, sensitization of health workers, women, and improving folic acid supply is recommended.

## Introduction

Spina Bifida is a neural tube defect (NTD) caused by a fault in the development of the central nervous system in the first 25 days of the pregnancy [1]. The worldwide incidence varies between 0.17 and 6.39 per 1000 live births [2–5]. An estimated 1,400 children are born with spina bifida in Uganda annually [6]. Incidence and prevalence rates in Uganda are probably higher due to absence of folate consumption by pregnant women, lack of pre-natal care [7], absence of secondary prevention [8], higher exposure to environmental risk factors such as dioxins [9] and fumonisins intake [10–12], and high birth rates [6].

Folic acid (also referred to as folate, folacin, Vitamin B9, pteroylglutamic acid), delivered through supplementation or fortification (or other strategies) prevents the first occurrence of NTDs [13, 14] as well as the recurrence of NTDs in families with previous NTD-affected pregnancy [15]. The World Health Organization (WHO) recommends that all women of childbearing age consume 400 µg of folic acid daily and that women with pregnancies previously affected by NTDs consume 5000 µg of FA daily [16]. Women should consume these amounts in the peri-conceptional period as it takes 8 weeks to reach the optimal level of serum folate [17]. Supplements are an effective way to prevent spina bifida, but there is low compliance [18, 19]. Countries fortifying the flour with at least folic acid see an average 46% reduction of NTD prevalence [20].

In Uganda only 17% of pregnant women attend antenatal care (ANC) before the fourth month of pregnancy [21]. Of those who do attend ANC women are 5.5 months pregnant (median) when they come for their first visit. The Ugandan Government advises intake of folic acid following WHO recommendations [22]. In a study in rural Uganda 13.2% of women attending ANC took folic acid during pregnancy [23]. The health indicators for the northern region are worse compared to the rest of the country [21], partly attributed to conflict between the Government of Uganda and the Lord's Resistance Army (1986 - 2006), which displaced an estimated 2 million people into internally displaced people (IDP) camps [24]. Lack of human resources, limited knowledge, shortages of medical supplies and inadequate infrastructure hamper implementation of quality health services [25, 26]. The study area, Gulu district had 410,673 inhabitants with 15,992 deliveries, 33% of pregnant women attending 4 ANC visits, and 72% of staff positions filled in health facilities in 2012 [27]. Uganda passed a national legislation to require fortification of wheat and maize flours with iron, zinc, folic acid and other B vitamins in 2011. The legislation requires fortification of white and brown wheat flours and maize products produced in Uganda as well as those imported to Uganda. Whilst some flour producers have started fortifying their products, not all are compliant [28].

## Methods

Quantitative and qualitative descriptive cross-sectional survey methods were employed to collect data on folic acid intake and understanding of its effect on the prevention of spina bifida in Gulu district. In total 15 mothers of children with spina bifida were asked about folic acid intake during their pregnancy. In addition 394 women in reproductive age group (15-49 years) attending ANC were surveyed. As well 35 health workers, working at ANCat government health facilities were interviewed. The 394 women and 35 health workers were selected by simple random sampling at antenatal clinics. The mothers of children with spina bifida were purposefully selected from a mobile specialist review clinic for children with neurological conditions in Gulu. The data collection tools included semi structured questionnaires and interviews. Data was entered and analyzed using SPSS16.

## Results

Study participants had a median age of 29 years, the majority was married, Christian, completed primary school, and had 3 or 4 children. Table 1 shows the demographic characteristics of 15 mothers of children with spina bifida, 394 women in reproductive age attending antenatal clinics, and 35 health workers. Of the 15 mothers of children with spina bifida interviewed, only a fourth took folic acid supplements during pregnancy, and none of them took these in the first semester (table 2). All mothers of children with spina bifida had never heard about spina bifida before they gave birth to their child, and had not known about the preventative effect of folic acid. All said they ate vegetables rich in folic acid such as spinach and avocado before and during pregnancy, but not on a daily basis. Intake varied during the year depending on whether they were dependent on food handouts during their time in the IDP camps, and/or the season and availability. None had heard about food fortification before having a child with spina bifida.

**Table 1:** Demographic characteristics respondents northern Uganda

Characteristics	Mothers of children with spina bifida (N = 15)	Women attending antenatal clinics (N = 394)	Health workers in antenatal clinics(N = 35)
**Age (mean)**	31 (range 22 – 36)	29 years (range 18 – 40, SD 7.8)	28 years (range 22 – 42)
**Marital status**	6.7% (1) single, 60% (9) married, 13.3% (2) separated, 20% (3) widowed	19% (75) single, 72.2% (284) married, 5% (20) separated, 3.8% (15) widowed	11.4% (4) single, 88.6% (31) married
**Number of children**	4.2 mean (range 1 – 8)	3.8 children (range 0 - 9)	3.1 mean (0 – 6)
**Education**	26.6% (4) never went to school, 60.0% completed primary (9), 6.7% (1) completed secondary, 6.7% (1) higher education	15.8% (63) never went to school, 55.6% (219) completed primary, 22.4% (88) completed secondary, 6.2% (24) higher education	54% (19) nursing - enrolled, 11% (4) registered nursing - registered, 35% (12) midwifery
**Religion**	86.7% (13) Christian 13.3% (2) Muslim	97% (382) Christian 3% (12) Muslim	98% (34) Christian 2% (1) Muslim
**Occupation**	73.2% (11) peasant farmers, 13.4% (2) petty traders, 13.4% (2) others	44.7% (176) peasant farmers, 39.9% (157) petty traders, 15.5% (61) others	54% (19) enrolled nurse, 11% (4) registered nurse, 35% (12) midwife

**Table 2:** Folic acid intake in mothers of children with spina bifida and women attending ANC in northern Uganda

Started taking FA	Mother of children with spina bifida	Women attending antenatal care
Before conception	0% (0)	0% (0)
During pregnancy (first trimester)	0% (0)	8.1% (32)
During pregnancy (2nd and 3rd trimester)	26.7% (4)	42.4% (167)
Did not take FA	(11)	49.5% (195)

Of the women attending ANC, 65.8% lived within 2 kilometers of the health facility (259), 23.4% (92) within 5kms, and 11% (43) beyond 5kms. 50.5% (197) took folic acid, none before pregnancy, and only 8.1% (32) during the first trimester of pregnancy (table 2). In total 33.5% (132/394) of the women attending ANC had ever heard about spina bifida. Only 1% (2) knew about the preventative effect of folic acid intake. Women who had knowledge about spina bifida were more likely to take folic acid than those who did not (table 3, p < 0.001). All women said they ate vegetables rich in folic acid such as spinach, okra, beans, lentils and avocado regularly. Intake varied during the year depending on the season and availability. None of the women had heard about food fortification or recognized the signs on available food products. Of the women taking folic acid, 75.5% (149/197) said folic acid (in 5mg tablets) was available at the health facility during their visits. The other 24.5% (46) said sometimes stock outs occurred. Those who had access to folic acid at the health facility were more likely to take it (p < 0.001). Women attending health education at the health facilities were more likely to take folic acid compared to those who did not receive health education (p < 0.001).

**Table 3:** Factors associated with folic acid intake in women attending ANC in northern Uganda

Factors		Folic Acid Intake			
		yes	No	N (total)	X^2^
Knowledge of spina bifida	Yes	91 (45.5)	41 (21.1)	132 (33.5)	26.24*
	No	109 (54.5)	153 (78.9)	262 (66.5)	
Availability of drugs	Yes	168 (87.0)	121 (63.7)	289 (75.5)	28.22*
	No	25 (13.0)	69 (36.3)	94 (24.5)	
Health education	Yes	52 (25.7)	13 (6.6)	65 (16.3)	26.59*
	No	150 (74.3)	183 (93.4)	333 (83.7)	

* p<0.001

All health workers interviewed had ever heard of spina bifida. The majority of the health workers, 80% (28/35), believed folic acid intake and good antenatal care could prevent spina bifida, 9% (3/35) believed there is no prevention, while 11% (4/35) were not aware of any. Of those who believed folic acid intake could prevent spina bifida (28/35), 7% (2/35) said it should be taken before pregnancy and early weeks of pregnancy, 25% (7/35) said preferably before birth or as soon as the woman realizes she is pregnant for a period of 90 days, 39% (11/35) recommended folic acid use (5mg tablets once a day) throughout pregnancy, and 29% (8/35) health workers recommended folic acid use in only the second and third trimester. All health workers felt that intake of folic acid was important for the babies general development, and mother's health. A fifth (7/35) of the health workers said folic acid is not always available at the health facility they work at. None of the health workers had heard about food fortification.

## Discussion

Our findings show that knowledge about the preventative effect of folic acid on spina bifida, and folate intake before and during the first weeks of pregnancy is very limited in northern Uganda. Awareness of spina bifida (33.5%) in women attending ANC was in line with earlier studies, e.g. 25.5% in Nigeria [29], and 53% in Congo [30]. With 17.3% of the Ugandan women in our study taking folic acid supplements during the first trimester of pregnancy, the percentage is slightly lower than the worldwide estimate of only 20% of pregnant women being able to follow the recommendation of folic acid intake for prevention [31]. Whilst peri-conceptional intake of folic acid supplements was 17.2% in Tanzania [32], our study population did not report any early intake of supplements. Women did report to regularly consume foods rich in folic acid. One of the limitations of our study is that it could not define the amounts consumed, as no food samples or biomedical investigations were conducted. The lack of folic acid intake in northern Uganda could partially be explained by the lack of knowledge on the importance of intake prior to and in the first 28 days of pregnancy amongst the health workers interviewed. Health workers in our study had knowledge about the importance of folic acid intake during pregnancy but not about the crucial pre-conceptual and early conceptual period, they were also not aware of the recommended dosage too.

Sensitization of health workers and women in reproductive age is key to encourage early ANC attendance and folic acid intake. Alongside sensitization at the antenatal clinic, women should receive sexual and reproductive health education from an early age. Following the conflict, educational attainment has been lacking in the northern region, and girls secondary school completion remains low [33]. Counseling on folic acid use in the media and marriage counseling may be considered to inform young couples as many will not have received formal sexual and reproductive health education. In Congo patient education through video media increased awareness and knowledge of spina bifida and folic acid [30]. In northern Uganda mobile video media could be explored. Radio messages have proven powerful in transferring health messages in the region and could also be used [34–36].

To improve access to folic acid supplements, there is need to strengthen the supply chain and address stock outs, so that folic acid supplements are provided at the first antenatal visit, or ideally to all women in child bearing age. Gulu district health facilities had a 57% basic drug stock out in 2012 [27]. Aside assuring folic acid stocks in health facilities, we recommend sensitization on the correct dosage. In a Uganda a huge difference was found between and within districts in the dosage of folic acid provided to pregnant women [37]. The differences were not only caused by lack of supplies but also by health worker's lack of knowledge on updated guidelines. During the war health structures in northern Uganda dilapidated and health workers in the area may not have received the necessary training, resulting in knowledge gaps. With targeted recovery plans of the Ugandan Government, supported by donors, and national guidelines from the Ministry of Health, the health system is recovering and targets are set to improve maternal and child health [22, 38, 39]. Whilst educating health workers on the correct dosage, supplies of 400mcg rather than 5mg tablets should be availed by the National Medical Stores to enable health workers provide the correct preventative dosage, as 1mg and not 5mg is the upper tolerable daily limit [40].

Together with improving folic acid supplementation, we argue for mandatory food fortification and implementation of this process at national level to increase levels of folic acid intake for all women in childbearing age. Participants in our study were not familiar with food fortification. Globally 25% of folic acid-preventable spina bifida is being prevented, and mandatory food fortification is recommended to achieve total prevention [41]. Sensitization about food fortification and recognition of the label can increase use of fortified foods. In South Africa folic acid fortification of staple foods reduced the prevalence of spina bifida with 41.6%, benefits outweighed the costs of food fortification [42]. It should be noted that not all women in reproductive age will benefit from food fortification, as many are subsistent farmers and are more likely to feed from their own produce than goods sold on the markets. We recommend further studies involving women prior to conception, during pregnancy and after birth with frequent measurements of folic acid intake to understand the actual levels of folic acid intake in the population, and evaluation studies to measure possible effects of sensitization campaigns and training of health workers in future.

## Conclusion

Folic acid intake is limited in northern Uganda. This is attributed to limited education and understanding of women and health workers about the importance of early folic acid intake, late presentation of women at ANC, poor supply chain and dilapidated health services caused by war and poverty. A combination of food fortification, sensitization of health workers, women, and improving folic acid supply is recommended to reduce the number of children born with neural tube defects such as spina bifida.
